# Choroid plexus epithelium and its role in neurological diseases

**DOI:** 10.3389/fnmol.2022.949231

**Published:** 2022-10-21

**Authors:** Ruizhen Liu, Zhiping Zhang, Yibing Chen, Junbo Liao, Yuchang Wang, Jingping Liu, Zhixiong Lin, Gelei Xiao

**Affiliations:** ^1^Department of Neurosurgery, Xiangya Hospital, Central South University, Changsha, China; ^2^Diagnosis and Treatment Center for Hydrocephalus, Xiangya Hospital, Central South University, Changsha, China; ^3^National Clinical Research Center for Geriatric Disorders, Xiangya Hospital, Central South University, Changsha, China; ^4^Department of Neurosurgery, Sanbo Brain Hospital, Capital Medical University, Beijing, China

**Keywords:** choroid plexus epithelium, Alzheimer's disease, Parkinson's disease, hydrocephalus, stroke, treatment

## Abstract

Choroid plexus epithelial cells can secrete cerebrospinal fluid into the ventricles, serving as the major structural basis of the selective barrier between the neurological system and blood in the brain. In fact, choroid plexus epithelial cells release the majority of cerebrospinal fluid, which is connected with particular ion channels in choroid plexus epithelial cells. Choroid plexus epithelial cells also produce and secrete a number of essential growth factors and peptides that help the injured cerebrovascular system heal. The pathophysiology of major neurodegenerative disorders like Alzheimer's disease, Parkinson's disease, as well as minor brain damage diseases like hydrocephalus and stroke is still unknown. Few studies have previously connected choroid plexus epithelial cells to the etiology of these serious brain disorders. Therefore, in the hopes of discovering novel treatment options for linked conditions, this review extensively analyzes the association between choroid plexus epithelial cells and the etiology of neurological diseases such as Alzheimer's disease and hydrocephalus. Finally, we review CPE based immunotherapy, choroid plexus cauterization, choroid plexus transplantation, and gene therapy.

## Introduction

The choroid plexus epithelium (CPE), a specialized compartmental membrane cell at the choroid plexus created by the ventricle wall, is a glial cell of the central nervous system (CNS). The blood-cerebrospinal fluid barrier (BCSFB) is maintained by the apical tight junctions of the CPE, which are made up of a layer of cuboidal to low cylindrical epithelial cells joined by contact complexes. Scientists have long been concerned about the CPE being the primary source of secreted cerebrospinal fluid (CSF). Despite a substantial amount of research on the CPE's robust ability to secrete CSF, the potential link between the CPE and diseases caused by a disrupted or dysregulated CSF ion balance, as well as the pathophysiology of these disorders, remains unknown (Redzic and Segal, 2004; Chodobski et al., 2019). At the same time, several of the active chemicals it produces, such as Insulin-like growth factor, suggest that it has a role in brain repair and inflammation (Ghersi-Egea et al., 2018). It's worth noting that the distribution of several chemicals in the CPE differs significantly from that of other epithelial cells, and it has a particularly appealing reverse polarity (Damkier et al., 2018).

CPE anomalies have been linked to the development of numerous neurological illnesses, including hydrocephalus and Alzheimer's disease (AD), according to recent research. Damage to the CPE will result in a lethal blow to the blood-brain barrier, which will have a significant impact on brain fluid circulation and may potentially lead to the development of hydrocephalus (Yamada et al., 2021). CPE failure is present in AD, and changes in cerebrospinal fluid production and CPE converging effects, as well as the lack of CPE-mediated macrophage recruitment, all contribute to AD pathogenesis (Mesquita et al., 2012).

This review summarizes recent research on choroid plexus epithelial cell malfunction and neurological illnesses, as well as the involvement of epithelial cell venation in neurological diseases. The relationship between the mechanism of these connected brain disorders and the aberrations of the CPE is thoroughly studied in this work, which describes numerous types of typical brain diseases such as neurodegenerative diseases. We want to encourage more research into choroid plexus epithelial cells as a target for neurological disease therapy and innovative targeted medicines.

## Choroid plexus epithelium

### Structure

**Figure 1:**
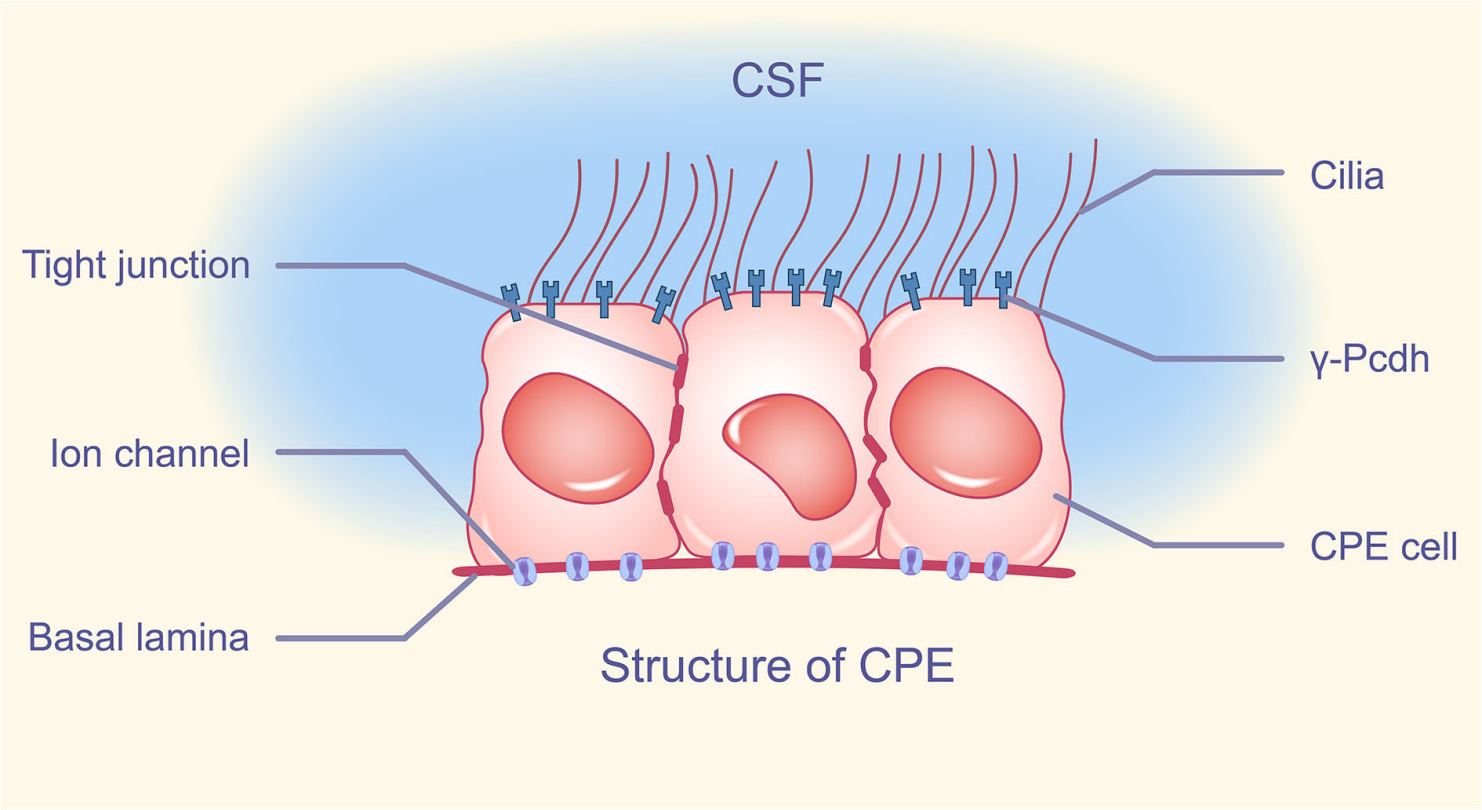
Structure of CPE. The apical membrane of CPE has a variable number of cilia, intercalated between cells, tight junctions composing BCSFB, and cell adhesion molecule γ-Pcdh distributed on the apical membrane except cilia. A large number of ion channels are distributed in the basement membrane of CPE. CPE, choroid plexus epithelium; BCSFB, blood-cerebrospinal fluid barrier; γ-Pcdh, Pcdh-γ gene family.

Through a thin layer of connective tissue and fenestrated capillaries in the equivalent bottom layer, blood equilibrates with the CPE. The CPE features a tight junction (TJ) nearest to the luminal membrane, which divides the ventricular lumen from the lateral intercellular and basal gaps, similar to other epithelia. Desmosomes appear to be more inferior to the adherens junction, which is positioned below the TJ. The surface of the CPE has many microvilli and many different kinds of motor cilia. Except for the transition zone between the lateral and basal zones, the membranes of the lateral and basal zones do not expand through surface extension. Adjacent cells create multiple interdigitating protrusions that form a basolateral maze at this location, resulting in a 10-fold increase in surface area (Cserr, 1971; Mortazavi et al., 2014; Spector et al., 2015; Praetorius and Damkier, 2017; Ghersi-Egea et al., 2018). Interestingly, there have been numerous reports on the heterogeneity of the CPE (Hirano et al., 2002; Lobas et al., 2012; Wang et al., 2019; Perin et al., 2021). For example, choroid plexus epithelial cells not only highly express most members of the Pcdh-γ gene family (γ-Pcdh) of cell adhesion molecules, but also γ-Pcdh expression is strictly restricted to the apical membrane and is not expressed in tight junctions, basolateral adherens junctions, and apical ciliary clusters (Lobas et al., 2012) (Figure 1).

### Function

Previous studies have revealed that the CPE, as an important structure in the brain, plays an important role in cerebrospinal fluid homeostasis balance (Husted and Reed, 1977; Kazemi and Choma, 1977; Lindsey et al., 1990; Bouzinova et al., 2005; Praetorius and Nielsen, 2006; Millar and Brown, 2008; Damkier et al., 2009, 2013, 2018; Christensen et al., 2017), neurogenesis (Baruch et al., 2014; Silva-Vargas et al., 2016), and inflammation regulation (Baruch and Schwartz, 2013; Baruch et al., 2013; Schwartz and Baruch, 2014).

The composition and acidity and alkalinity of cerebrospinal fluid maintain a dynamic balance under normal physiological conditions and play a unique role in regulating important life activities such as inflammation regulation. This maintenance of homeostasis is inextricably linked to some important ion transporters or anion transport channels on the CPE (Damkier et al., 2018), such as acid-base transporters Cl^−−^/H^+^, NHE6 and CLC-7 on the CPE (Kazemi and Choma, 1977; Damkier et al., 2018). The distribution of these transporters on the CPE can be remarkably heterogeneous (Lobas et al., 2012; Ghersi-Egea et al., 2018), which correlates with their function. There are also many microvilli and active cilia on the surface of the CPE, which play an important role in cerebrospinal fluid dynamics and can ensure the normal transport and circulation of cerebrospinal fluid (Cserr, 1971). If the cilia are damaged, retention of CSF may lead to hydrocephalus and other diseases (Oi et al., 2011; Kumar et al., 2021).

The first report of cation channel gene disruption leading to CPE dysfunction was published in 2013 (Roepke et al., 2011), and suggests that KCNE2 regulates KCNQ1 and KCNA3 in the CPE to influence blood-CSF anion flow. In the choroid plexus epithelium, KCNE2 forms potassium channels with KCNA3 and KCNQ1. In addition to transporters and channel proteins for diverse solutes and water, glucose, fructose, and uric acid transporters have been discovered in choroid plexus epithelial cells, which could serve as neuroprotective energy and antioxidation substrates.

Choroid plexus epithelial cells in the lateral ventricle not only directly contact adult neural stem cells in the ventricular-subventricular zone in the CSF, but also secrete cellular activation and proliferation factors that promote the development of adult neural stem cells in the ventricular-subventricular zone in the lateral ventricle choroid plexus epithelial cells, and these brain microecological factors play an important role in the neural development of adult neural stem cells in the ventricular-subventricular zone throughout the life cycle (Silva-Vargas et al., 2016). The CPE separates CSF from tissues within the choroid plexus (CP) such as capillaries. Under normal physiological conditions, the CPE maintains CSF homeostasis by secreting neurotrophins and regulating pH. When chronic neuroinflammation occurs, leukocytes need to be recruited from peripheral tissue vessels and enter the central nervous system through the “gate”. Recent studies have confirmed that the blood-cerebrospinal fluid barrier is a selective channel for leukocyte entry during inflammation (Schwartz and Baruch, 2014). The CPE promotes leukocyte recruitment and trafficking by expressing chemokines and adhesion molecules that support transepithelial leukocyte migration (Kunis et al., 2013; Schwartz and Baruch, 2014). Direct evidence of migration of leukocytes such as T cells, neutrophils, etc., has also been found in the CPE during inflammation (Carrithers et al., 2000; Szmydynger-Chodobska et al., 2012). The CPE is also involved in the accumulation of inflammatory cells in the brain following stroke, which will be detailed in Section Stroke below.

## Neurological diseases related with the choroid plexus epithelium

In today's world, neurological problems are extremely common, especially among the elderly (Chan et al., 2013). Most chronic neurological illnesses have a complex etiology that has yet to be determined. However, central nervous system inflammation is linked to dysregulation as people age (Skaper et al., 2018). The CPE plays an essential role in the regulation of brain homeostasis as the main organ secreting CSF in the brain, and current studies suggest that the CPE is a portal for leukocytes to enter the brain (Schwartz and Baruch, 2014). The CPE also plays an important role in the pathogenesis of the central nervous system (Bolos et al., 2013; Barbariga et al., 2020). Accelerated CPE atrophy has been reported in stroke, multiple sclerosis, schizophrenia, and other central nervous system illnesses (Silverberg et al., 2003; Redzic et al., 2005; Schmidt et al., 2005), indicating that CPE atrophy is linked to CNS disease aggravation and that ciliary abnormalities on the CPE are linked to hydrocephalus (Narita et al., 2010). In the central nervous system, there is only one main thyroid hormone carrier partitioning protein: CP-derived transthyretin. As a result, a lack of thyroid hormone in the central nervous system is linked to aberrant brain development, adult dementia, depression, and other cognitive issues (Davis et al., 2003). The pathogenesis of AD is linked to three major methods of amyloid-β (Aβ) peptide clearance: cerebrospinal fluid-mediated A clearance, direct Aβ absorption, and synthesis of Aβ partners and proteases, all of which may contribute to impaired Aβ clearance in our brains. Alzheimer's disease is also linked to CPE atrophy (Serot et al., 2003; Silverberg et al., 2003). As previously stated, the CPE serves as a conduit for the recruitment of leukocytes from distant places into the brain, and the incidence and treatment of many CNS inflammations are inextricably linked to the CPE's effect on inflammatory cells. Based on the important role of the CPE in CSF secretion, regulation of brain homeostasis, development of the central nervous system, and regulation of chronic inflammation in the central nervous system, the possible mechanism by which CPE abnormalities lead to neurological diseases will be discussed later.

### Hydrocephalus

Hydrocephalus is a common brain disease in which the amount of intracranial CSF increases, the ventricular system enlarges, or the arachnoid membrane enlarges due to excessive CSF secretion or circulation and malabsorption caused by craniocerebral diseases. The mainstream classification divides hydrocephalus into obstructive hydrocephalus and communicating hydrocephalus. Obstructive hydrocephalus, also known as non-communicating hydrocephalus or intraventricular obstructive hydrocephalus, which refers to the formation of lesions located in or near the ventricular system and the blocking the cerebrospinal fluid circulation in the ventricular system. Hydrocephalus caused by obstruction above the exit site of the fourth ventricle is the most common form of hydrocephalus. It is common in arachnoid cysts, aqueductal atresia or stenosis, and dysplasia of the median or interventricular foramen. Communicating hydrocephalus, on the other hand, is hydrocephalus due to an obstruction of the extraventricular cerebrospinal fluid circulation pathway or CSF malabsorption and can also be caused by excessive cerebrospinal fluid secretion such as choroid plexus papilloma (Oi et al., 2011; Li et al., 2021).

The basic causes of hydrocephalus are divided into three types: Obstruction, CSF malabsorption, and excessive CSF production. Hydrocephalus may occur if obstruction occurs in any part associated with normal CSF flow. CSF malabsorption is usually caused by inflammation of the brain tissue, and of course CPE ciliary abnormalities can also cause congenital hydrocephalus (Oi et al., 2011). Excessive CSF production may manifest as diffuse villous hyperplasia of the choroid plexus (Smith et al., 2007; Kumar et al., 2021). In addition, central nervous system infection and cerebral hemorrhage can be the origin of hydrocephalus (Karimy et al., 2017).

As a common type of hydrocephalus, posthemorrhagic hydrocephalus (PHH) is a condition that occurs frequently in premature infants. The current understanding of PHH is still in its early stages, and CSF drainage is also basically used to treat PHH, but the optimal timing of intervention is not clear. Recent studies have revealed a potential link between PHH and the CPE that has attracted our attention. In a rat model established with PHH, the investigators demonstrated that intraventricular hemorrhage (IVH) leads to toll-like receptor 4 (TLR4) and nf-kb-dependent inflammatory responses in the CPE. The IVH-induced CSF hypersecretion is mediated by TLR4-dependent activation of the ste20-type stress kinase SPAK, which binds, phosphorylates, and stimulates NKCC1 co-transporters on the apical membrane of the CPE. Genetic deletion of TLR4 or SPAK can normalize the rate of hyperactive cerebrospinal fluid secretion and reduce PHH symptoms (Karimy et al., 2017). Similarly, 5-chloro-N-(5-chloro-4-((4-chlorophenyl)(cyano)methyl)-2-methylphenyl)-2-hydroxybenzamide (ZT-1a) administration has been found to reduce inflammation-induced cation-Cl^−^ cotransporters (CCC) phosphorylation of CP and CSF oversecretion in a model of PHH (Zhang et al., 2020). This reveals a direct association between CSF hypersecretion due to altered ion transport mechanisms in CPE and hemorrhagic hydrocephalus. At the same time, more and more recent studies have shown that there is an inseparable link between cerebrospinal fluid composition and the occurrence of PHH. Cerebrospinal fluid NCAM-1 concentration was shown to be associated with neurodevelopmental outcomes in premature infants with hemorrhagic hydrocephalus (Limbrick et al., 2021), and cerebrospinal fluid amyloid levels in premature infants with hemorrhagic hydrocephalus were also shown to be associated with ventricular size (Morales et al., 2015).

Choroid plexus papilloma is a rare central nervous system tumor more common in children, accounting for <1% of brain tumors. After surgical treatment, the mortality rate is <1%. Even so, choroid plexus papillomas are associated with hydrocephalus. Many case reports have been recorded (Sahar et al., 1980; Kuyama et al., 1987; Miyagi et al., 2006). However, whether choroid plexus papillomas can cause hydrocephalus or whether there is a causal relationship between the two is still unsatisfactory. Tumor-induced hydrocephalus may result from excessive CSF secretion or ventricular blockage. Studies have shown that AQP1 expression is strong and diffuse in low-grade choroid plexus papillomas, but not in high-grade choroid plexus papillomas, which may be associated with the disappearance of papillary structures in high-grade tumors. And in low-grade choroid plexus papillomas, AQP1 expression had no significant polarity, no clear expression pattern, and each patient had a significant difference, and AQP1 expression on the normal choroid plexus had a significant polarity completely opposite, but this study was unattended (Longatti et al., 2006). Alternately, studies have concluded that CSF secretion is not significantly increased in choroid plexus papillomas (Sahar et al., 1980). These studies suggest that choroid plexus tumors may not be associated with CSF hypersecretion. Perhaps it is only the tumor that blocks the ventricles that leads to hydrocephalus. We expect future studies to overturn or prove this view.

In brief, when there are CPE tumors in the brain, changes in the CPE ion transport mechanism, CPE ciliary malformation, CP hyperplasia, abnormal CSF composition, etc., it will have a great impact on the normal absorption and excretion of CSF, CSF circulation, and finally lead to the occurrence of hydrocephalus.

### Stroke

Stroke is a collective term for acute cerebrovascular diseases in traditional Chinese medicine. Stroke is a common brain disease in which brain tissue injury is caused by sudden rupture of blood vessels in the brain or by ischemia of the brain due to blockage of blood vessels. Many times, stroke is the result of cerebral ischemia and it is more common in the elderly. For children, arterial infection is the main cause of stroke (Fullerton et al., 2015, 2017). Irreversible damage to brain tissue by stroke can lead to the death of a large number of brain cells within a few minutes, which is a great damage to neurological function, and leads to disability (Hornung and Sievert, 2021). Stroke is currently one of the diseases of high concern all over the world. Since stroke is an acutely developing serious brain disease, patients must be sent to the hospital as soon as possible. For stroke patients, time is life (Davies and Delcourt, 2021).

At present, the etiology of stroke is mainly about cerebrovascular artery occlusion or cerebrovascular, thrombotic bleeding. For the generation of cerebral thrombosis, the most important and direct factor is cerebrovascular lesions such as atherosclerosis. Poor life habits such as smoking, obesity, and hypertension are important inducers of cerebral thrombosis and are positively correlated with the production of cerebral thrombosis (Ozturk, 2014). The CPE does not have direct contact with blood, abnormalities in the CPE do not appear to be directly related to thrombus, and abnormalities in the CPE do not appear to directly contribute to stroke. However, cases of an abnormal CPE are often present in hypertensive patients (Ruchoux et al., 1992) and hypertension is an important potential factor for stroke. At the same time, the CPE plays an important role in the mechanism of brain cell injury after stroke (Llovera et al., 2017). A profound understanding of these two points will greatly promote the treatment of stroke.

Hypertension is the most common chronic disease among the elderly, primarily essential hypertension. Studies have found that in spontaneously hypertensive mice, the CPE nuclei show heterogeneous chromatin. The number of Golgi apparatus of an abnormal CPE was higher, mitochondria were also more prevalent, microvilli were significantly lost, and basement membrane infoldings were reduced (Ruchoux et al., 1992). It was then found that the expression of the natriuretic peptide A receptor and cGMP production were reduced in the choroid plexus of spontaneously hypertensive rats (Zorad et al., 1998). Later studies of the CP in the brain of stroke-prone spontaneously hypertensive rats showed that activation of the MR/ENaCS pathway of the CP in stroke-prone spontaneously hypertensive rats is involved in the development of hypertension through elevation of CSF (Nakano et al., 2013). These results suggest that there should be similar changes in the CPE in hypertensive patients.

Although brain cell injury obviously occurs after stroke, in addition to ischemia itself, neuro inflammatory brain injury is more studied. There are many studies on the convening of inflammatory cells and the invasion of brain cells after stroke, but the role of the CPE as a BCSFB in this process is not particularly clear. Some studies have reported the specific accumulation of T cells in the peri-infarct cortex and detected T cells as the major population in the ipsilateral CP in mice as well as in post-stroke autopsy samples in humans (Llovera et al., 2017). *In vivo* cell tracking of photo activated T cells confirmed the migration of T cells from the CP to the peri-infarct cortex (Llovera et al., 2017). Neonatal stroke and TLR1/2 ligands have also been found to recruit myeloid cells through the choroid plexus *via* CX3CR1-CCR2 and context-specific manners (Rayasam et al., 2020). And the response of the CP to cortical stroke was achieved by up-regulating the gene expression of monocyte-derived macrophage transport mediators, as shown by the increase of monocyte-derived macrophages in the CP and CSF (Ge et al., 2017). A full understanding of the invasion of brain cells by neuro-inflammatory cells after stroke by the CPE can facilitate the development of corresponding therapeutic measures for the CPE in the future to reduce the damage to brain cells after stroke and greatly increase the sense of accomplishment of stroke treatment.

In conclusion, an abnormal CPE and hypertension often appear together, and hypertensive patients with an abnormal CPE are prone to stroke, and the causal relationship between an abnormal CPE and hypertension needs to be further demonstrated. Secondly, the CPE plays an important role in the mechanism of brain destruction by post-stroke inflammation. The CPE is involved in the accumulation of inflammatory cells in the post-stroke brain and is a potential therapeutic target to reduce post-stroke brain injury.

### Alzheimer's disease

Alzheimer's disease (AD) is the most common neurodegenerative disease of the central nervous system in elderly or pre-elderly patients. The language, emotion, cognition, and calculation of the elderly with AD can be devastated. At present, there is basically no possibility of cure, treatment is only to delay the disease; Alzheimer's disease is as irreversible as aging itself. The most striking abnormality in the patient's brain tissue is amyloid plaques with fibrillar tangles in neurons, mainly due to the deposition of amyloid beta and microtubule-associated tau. The current understanding of the etiology of AD is that protein polymers formed by excessive production or decreased clearance of beta-amyloid disrupt brain function. And hyperphosphorylation of tau affects the stabilization of neuronal skeleton microtubules and disrupts the normal function of neurons. But the understanding of why amyloid beta deposition with microtubule-associated tau occurs is not clear. Protein deposition and fibrillary tangles cannot be simply attributed to aging, because although the elderly have senile plaques, many of the elderly will not suffer from AD in their whole life. In recent years, the world has invested a lot in AD research, but still an effective means of cure has not been found. These results indicate that the pathogenesis of AD is complex and cannot be produced by impaired function of just one gene mutation. Recently, it was found that there is also β-like deposition in the CPE of AD patients, which may be associated with neuro inflammatory signals (Bolos et al., 2013). At the same time many have proposed that AD is associated with a decreased ability of the CPE to clear Aβ-like deposits (Serot et al., 2012). This indicates that there is a potential relationship between the occurrence of AD and the abnormality of the CP and aging (Serot et al., 2003; Bergen et al., 2015), but this aspect is often under appreciated.

The CPE acts as the main cell secreting CSF, but also secretes a variety of cytokines such as neurotrophic factors, which are also components in the CSF. Recently, a large number of studies have shown that aging and abnormalities in the CPE manifested as reduced tight junctions, reduced CSF secretion, and decreased ability to remove waste products (Brkic et al., 2015). Altered expression of several transporters such as lipoprotein receptor-related proteins 1 and 2 (LRP-1 and 2) are closely linked to AD (Balusu et al., 2016). Specifically, the expression of LRP-1 was down-regulated in AD patients compared with non-AD patients, and LRP-1 could promote the clearance of Aβ in AD patients (Serot et al., 1997).

Recently, it was found that choroid plexus annexin A5 levels were decreased in AD patients in the late stage of the disease, accompanied by increased Aβ levels and cell death, and that CSF annexin A5 levels were increased, indicating that annexin A5 is able to protect choroid plexus cells from Aβ-induced autophagic injury and apoptosis (Bartolome et al., 2020). It has also been proposed that the accumulation of tau and ApoE within the choroid plexus can increase the oligomerization rate of Aβ, which will impair the trafficking of tau, leading to pathological changes in AD (Raha-Chowdhury et al., 2019). These results indicate that Aβ deposition in the CPE of AD patients has a promoting effect on AD. According to the latest theory, AD is thought to be caused by chronic neuroinflammation. Recently, the CSF levels of the inflammatory cytokine TNF-α have been found to be significantly higher in AD patients than in non-AD patients, which may lead to the activation of matrix metalloproteinases (MMP) and lead to opening of the blood-cerebrospinal fluid barrier (Tarkowski et al., 2003). The above shows that the role of neuroinflammation on AD is not negligible.

In summary, several possible mechanisms of CPE abnormalities and AD are proposed: (1) Factors such as increased Aβ deposition in the CPE block the secretion of CSF, and the small flow of CSF substantially reduces the clearance of Aβ protein deposition in AD (Serot et al., 2012; Raha-Chowdhury et al., 2019). (2) The tight junctions of the CPE are greatly reduced, which increases the permeability of blood cerebrospinal fluid, allows inflammatory cells to enter, and causes an increase in the degree of chronic neuritis (Brkic et al., 2015). (3) Certain proteins (LRP-1) in the CPE can protect the CPE from harmful substances such as Aβ, but the reduced expression of this “beneficial protein” predicts the irreversible period (late) arrival of AD (Serot et al., 1997).

### Parkinson's disease

Parkinson's disease (PD) is a common neurodegenerative disease. The main pathological findings are resting tremor and muscle rigidity, and slow movement. It is more common in the elderly, and there are rare cases in adolescents. The causes of PD are not well understood, and current research tends to include multiple factors of genetics and aging contributing to the pathogenesis (Elbaz et al., 2016). The current mainstream understanding of its pathogenesis is that reduced DA synthesis in PD patients leads to reduced striatal dopamine (DA) content causing a series of symptoms including resting tremor, so the most classical drug for the treatment of PD is DA itself (Starkstein et al., 2012; Fernandez-Sotos et al., 2019).

Although the cause of PD is unknown, there are many factors that have been studied in relation to the pathogenesis of PD, and the CPE is a relatively clear target. In Parkinson's disease, the ferroxidase ceruloplasmin (Cp) is oxidized and deaminated by pathological CSF (Barbariga et al., 2020). In studies of human primary choroid plexus epithelial cells, the oxidized/deamidated-Cp was found to be an *in vivo* target for pathological CSF production, which mediates cell adhesion through isoDGR/integrin binding, transduces intracellular signals, and inhibits cell proliferation (Barbariga et al., 2020). There are also studies that have examined the ability of choroid plexus epithelial cell conditioned medium (CPEC-CM) alone or in combination with knockout serum (KS) to induce dopaminergic differentiation of human adipose-derived stem cells (hADSCs). The experimental results show that CPEC-CM and KS can act synergistically and significantly enhance the dopaminergic induction of hADSCs, which is exciting for the study of PD (Boroujeni et al., 2017).

The study of Parkinson-related gene Parkin co-regulated gene (PACRG) showed that PACRG was strongly expressed in the choroid plexus of the lateral ventricle, third ventricle, and fourth ventricle in mouse brains. Immunohistochemical analysis showed that PACRG is a component of ependymal cells and intraventricular cilia, and the results showed that PACRG is a component of ependymal cilia and plays an important role in the stability of the central nervous system (Wilson et al., 2009), This study showed a profound relationship between the CPE and PD. The CPE plays an irreplaceable role in the stabilization of the central nervous system. When an abnormal CPE occurs, it leads to a variety of serious diseases including PD. In the treatment of diseases such as PD, it is inevitable to think of whether the replacement of the CPE in patients will improve the disease, that is, choroid plexus transplantation. The experimental study of microencapsulated neonatal porcine choroid plexus cells in a non-human primate model of Parkinson's disease has emerged (Luo et al., 2013). However, in a recent follow-up study of PD patients for up to 104 weeks, no significant improvement in neurological function was observed in advanced PD patients treated with xenografted immune protective (sodium alginate-encapsulated) porcine choroid plexus cells (Mulroy et al., 2021). Although this result of is somewhat disappointing, it also reflects a problem that, for patients with advanced PD, it is not a simple change in the CPE function, but also accompanied by some irreversible changes in brain components (such as CSF components) and functions or mutations in genes, showing the complexity of PD.

In conclusion, the potential link between PD and the CPE has been confirmed by many studies so far (Wilson et al., 2009; Mulroy et al., 2021), but there is no clear answer. Alterations in CSF protein composition, possibly due to alterations in the CPE, contribute to the development of PD (Mulroy et al., 2021), but the causal relationship between the two needs to be further confirmed.

### Multiple sclerosis

Multiple sclerosis (MS) is a disease in which the myelin sheath of central nervous cells is damaged resulting in a series of adverse consequences including visual problems, sensory disturbances, and muscle weakness, which occurs frequently in whites, and is more common in women than in men (Wilson et al., 2009). It is not fully understood for the etiology of MS, but there have been related theories proposed such as destruction of the immune system and invasion of inflammatory cells into the brain leading to demyelination of nerve cells (Baecher-Allan et al., 2018).

Certain parenchymal changes in the CPE in the brains of MS patients, including reduced expression of claudin-3, a tight junction selectively expressed by the CPE, which increases the permeability of BCSFB, which may be an important channel for MS inflammatory cells to invade into the brain (Ricigliano et al., 2021). Studies have shown that in the brains of patients with multiple sclerosis, there are a large number of HLA-DR immune stained T lymphocytes and surface myocytes in the CPE, indicating that the CPE is actively involved in antigen presentation (Baecher-Allan et al., 2018). Memory B cells present in the CSF can enter the brain through the blood-brain barrier and BCSFB. Recently, a team has illustrated the important barrier role of BCSFB as a harmful activated B cell into the brain by screening the transcriptome of intrathecal B cells from MS patients through a series of cytokines/chemokines that mediate B cell diapedesis including qRT-PCR (Haas et al., 2020), while the permeability change of BCSFB is due to a series of changes including changes in the CPE. More hypoxia of CPE in the brains of MS patients than in normal subjects illustrates that the secretion of CPE undergoes non-negligible changes under MS pathological conditions (Rodriguez-Lorenzo et al., 2020).

**Figure 2:**
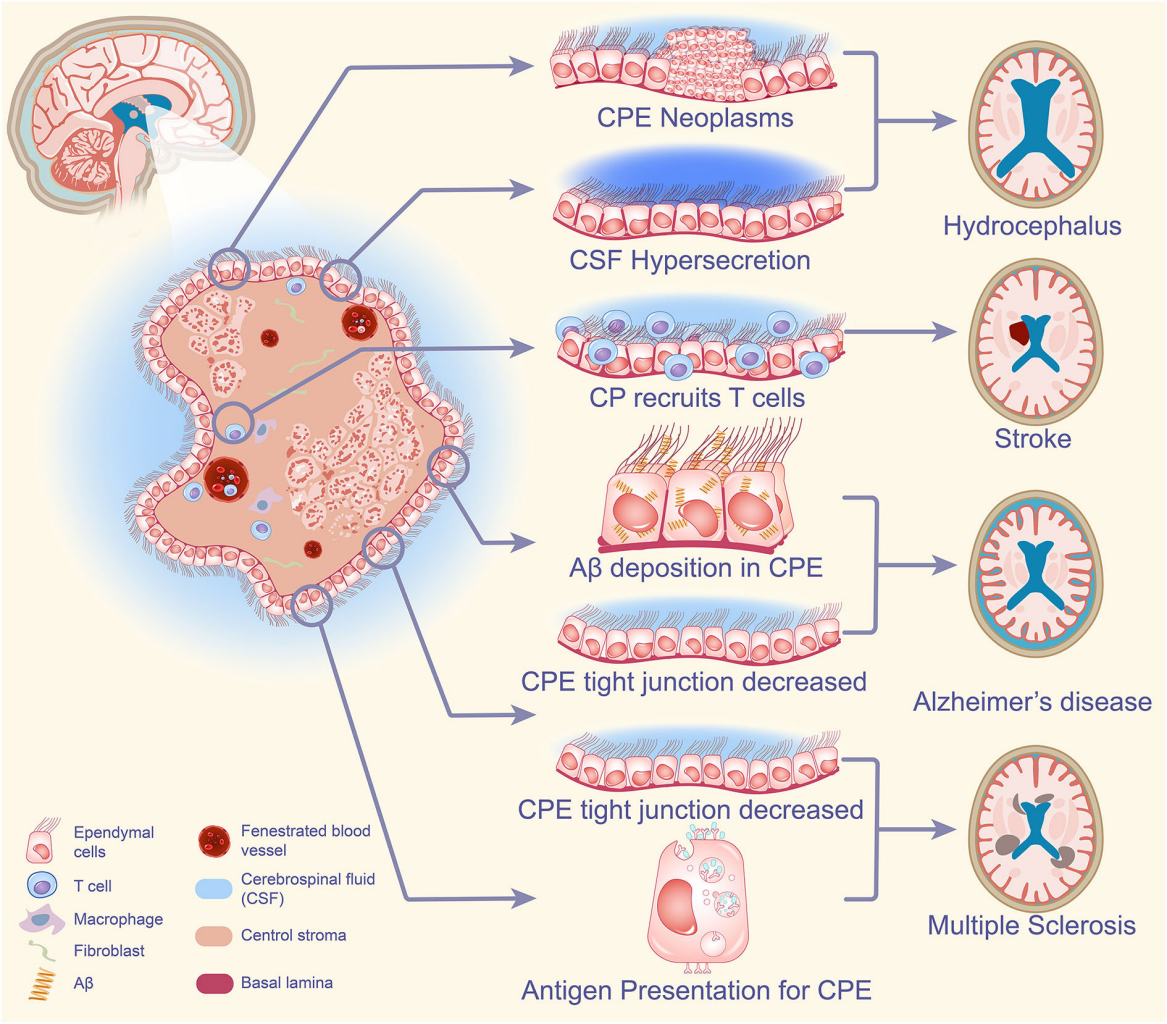
CPE-based neurological pathogenesis. When CPE tumors are present in the brain, excessive CSF secretion can have a great impact on the normal absorption and excretion of CSF, CSF circulation, and ultimately the development of hydrocephalus. The CPE is involved in the accumulation of inflammatory cells in the brain after stroke and plays an important role in the destruction of brain mechanisms by post-stroke inflammation. Increased Aβ deposition in CPE blocks the secretion of CSF, and the small flow of CSF significantly reduces the clearance of Aβ protein deposition in AD, while the reduction of tight junctions in CPE increases the permeability of blood cerebrospinal fluid, causing an increase in the degree of chronic neuritis, leading to the development of AD. Under the influence of peripheral neuroinflammation, the CPE changes and cellular tight junctions decrease, resulting in increased permeability of BCSFB and accelerated entry of inflammatory cells such as leukocytes into the brain to accelerate their injury. The CPE is also involved in the immune response process including antigen presentation and plays an important role in the pathogenesis of MS. CPE, choroid plexus epithelium; CSF, cerebrospinal fluid; Aβ, amyloid-β; AD, Alzheimer's disease; BCSFB, blood-cerebrospinal fluid barrier; MS, multiple sclerosis.

To conclude, under the influence of peripheral neuro inflammation, the CPE undergoes changes including reduced expression of claudin-3, which leads to increased BCSFB permeability, resulting in accelerated entry of inflammatory cells such as leukocytes into the brain to accelerate their damage, and the CPE is also involved in the process of these immune responses including antigen presentation (Ricigliano et al., 2021). The above is sufficient to illustrate that the CPE plays a non-negligible role in MS (Figure 2).

## Treatment

Through the above description, we can understand the key role of the CPE on the occurrence of neurological diseases. Nowadays, there are also many therapeutic measures targeting the CPE to treat neurological diseases. Some popular related therapeutic measures will be summarized below, in hopes of bringing us some inspiration.

### Immune therapy

In neurological diseases, the purpose of implementing immunotherapy is to intervene in certain immune links so as to prevent disease progression in autoimmune diseases such as MS. Interferon, the earliest discovered cytokine, is a glycoprotein produced by monocytes and lymphocytes and has a wide range of antiviral effects. At the same time, as the most first-line drug for the treatment of MS, its immunomodulatory effect is gradually moving toward the center. Interferon-α (IFN-α) and interferon-γ (IFN-γ) were used to treat MS in the earliest clinical trials, which significantly reduced the recurrence rate of patients and achieved preliminary results. But it was eliminated due to its serious side effects (Knobler et al., 1984; Camenga et al., 1986; Panitch, 1987; Durelli et al., 1994).

Finally, the low side effects and significant efficacy of IFN-β make it the most popular drug for the treatment of MS. As the first immune modulator approved for the treatment of relapsing remitting MS, it is divided into three different IFN-β formulations for its use, including “low-dose” IFN-β1a (Avonex) and “high-dose” IFN-β1a (Rebif22/44) or IFN-β1b (Betaferon, Betaseron). These agents are considered first-line disease modifiers for the treatment of MS (Kieseier, 2011). IFN-β treatment results in reduced magnetic resonance imaging (MRI) lesion activity, reduced relapse rates, reduced odds of brain atrophy and progression of persistent disability in MS patients. The beneficial effect of interferon-β (IFN-β) is thought to stem from its change in the environment from inflammatory regulation to regulation at several levels of immune cell activation, while preventing immune cell migration through the blood-brain barrier to the central nervous system. The pleiotropic effects of IFN-β on the peripheral immune system mainly include the reduction of pathogenic Th1 and Th17 cells and the increase of IL-10-producing Tregs *via* the JAK-STAT signaling pathway (Furber et al., 2017; Maimaitijiang et al., 2019; Ayatollahi et al., 2020). In addition, IFN-β reduces CD27^+^ memory B cells and increases IL-10-producing B cells, and IFN-β may downregulate the ability of adhesion molecules to inhibit pro-inflammatory cell entry into the CNS. These are sufficient to show that the therapeutic effect of IFN-β on the disease is multifaceted and effective.

As a CSF-producing organ, the immune monitoring of the CP on the brain and the repair after injury have also been studied a lot recently. Epithelial v-like antigen (EVA) found in mouse models is expressed in the CPE and CD4^+^ T lymphocytes and regulates CD4^+^ T lymphocyte adhesion to the CPE *in vitro* (Wojcik et al., 2011). Detecting the migration of T lymphocytes into the brain and the CP after enterotoxin-induced systemic immune activation showed that T lymphocytes preferentially migrated to the CP rather than to the brain (Petito and Adkins, 2005), showing a portal effect of the CP on T cells, which has been discussed above. Moreover, immune cells in the CP appear to communicate with epithelial cells through pannexin-1 channels expressed by epithelial cells in the CP, and excitedly, *in vitro* studies have shown that the expression of immune cell trafficking determinants in choroid plexus epithelium is specifically induced by IFN-γ (Kunis et al., 2013). *In vivo*, IFN-γ-dependent signaling controls transport mechanisms through the choroid plexus; IFN-γ receptor knockout mice have reduced levels of T cells and monocytes in the cerebrospinal fluid, and recovery after spinal cord injury is also impaired in mice. Moreover, reduced expression of choroid plexus trafficking molecules positively correlated with decreased CD4^+^ T cells in the choroid plexus and cerebrospinal fluid of IFN-γ receptor knockout mice. Similar effects were found in bone marrow chimeric mice lacking IFN-γ receptors in the central nervous system. Moreover, the experimental results also showed that the expression of TNF-α and IFN-γ receptors was mutually controlled by both through the CP (Kunis et al., 2013). This shows that for immunotherapy of MS, the exertion of a part of the effect of interferon depends on the CP. In other words, if the CP has some problems, it may affect the efficacy of interferon.

Therefore, for MS, an autoimmune disease, specific blockade of IFN-γ signaling pathway in the CP (add IFN-γ receptor antagonist or anti-IFN-γ antibody) may improve the development process of MS (Kunis et al., 2013). However, blocking signaling will lead to impaired recruitment of immune cells in the brain, which may lead to difficult recovery of inflammation in patients with CNS inflammation, so the indications for the disease require further study.

### Gene therapy

Gene therapy can treat diseases by exogenous input of genes (DNA, RNA) or intervention of genes resulting in their inability to translate and delete this gene normally, or manual editing of genes to delete the wrong gene and introduce normal genes. It has potential therapeutic value for congenital diseases. How to deliver gene modification-related small molecules or genes themselves for gene therapy is one of the major problems that plague gene therapy. There are now modes of viral delivery, non-viral delivery as well as cell delivery. Direct delivery was also often used early in the study, but this approach is clearly difficult to apply to humans. For targeted CP for gene therapy, the mode of its delivery has excitedly seen great possibilities in the current study.

Six different serotypes of adeno-associated virus (AAV) vectors (AAV2/1, AAV2/5, AAV2/8, AAV2/9, AAV2-BR1, and AAV2-PHP.EB) and lentiviruses have been injected into adult mice, respectively. It was found that AAV2/5 and AAV2/8 were significantly infected in the CP, and AAV2/1 was co-infected with ependymal cells in the CP. Transthyretin immunohistochemical staining indicates that AAV2/5 has a strong infectious capacity for the CPE and can act as a specific viral vector for the CP (Chen et al., 2020). While phages target epidermal growth factor, they are able to enter choroidal epithelial cells cultured *in vitro*, as tissue explants, or into viable choroidal epithelial cells by intraventricular injection. Gene expression in the choroid plexus and ependymal epithelium was detected by immunohistochemistry and non-invasive imaging when the phage genome was reconstituted into genes containing green fluorescent protein under the control of the cytomegalovirus promoter. Experiments have shown that recombinant ligand-mediated gene delivery is a feasible strategy that can improve the specificity of gene delivery to the central nervous system, and the CP is an important vector for this process (Gonzalez et al., 2010). Studies in which recombinant AAV serotype 5-green fluorescent protein was injected into the ventricles of pregnant mice also demonstrated that the CPE could act as a specific receptor for AAV5, with a sustained role (Haddad et al., 2013). At present, not many gene therapy studies have been conducted in the CP. Intracerebroventricular injection of adeno-associated virus type 5 (AAV5) containing reduced-size human ATP7A and copper chloride into neonatal mottled-brindled mice successfully rescued mice and greatly improved mouse survival. This demonstrates the important clinical value of gene therapy *via* the CP in the treatment of Menkes' disease (Donsante et al., 2011).

For the methods of virus administration in gene therapy, some articles have reviewed this (Jang and Lehtinen, 2022), and several methods of intravenous administration such as ultrasound-guided intrauterine vena cava administration have been proposed. The hazards of adenovirus use are also described in this article. In order to avoid the immune response produced by AAV, we can design a short DNA oligonucleotide that antagonizes toll-like receptor 9 (TLR9) activation and inserts it into the vector genome. We know that in AAVs, the vector genome can activate TLR9, TLR9 can recognize exogenous DNA (Chan et al., 2021), so the design can reduce innate immunity and T cell response.

In summary, targeting the CP for gene therapy is feasible. We can target and transmit certain effective therapeutic genes through the specific recognition of the CP by AAV5, which has very important clinical significance for neurodegenerative diseases of the brain or some refractory diseases. Of course, whether gene therapy by targeting the CP with AAV5 has significant side effects remains to be examined. Further research is needed on the injection of AAV5 into the brain itself. And what brain diseases do this gene therapy work? Which do not? These questions need to be studied more deeply.

### Choroid plexus cauterization

As the primary site of CSF production, the role of the CP is evident. However, for some conditions, such as communicating hydrocephalus, the balance between CSF secretion and absorption is broken so that CSF retention in the ventricles results in hydrocephalus. For its treatment, it is natural to start from the source, that is, to destroy the choroid plexus. However, as an important organ of the human body, there are indeed many unknown factors for directly burning or removing it. In 1910, when urologist Victor Darwin Lespinasse performed the first choroidectomy, the history of choroid plexus cauterization (CPC) began (Grant, 1996).

Theoretically, hydrocephalus should be relieved after CPC, however the clinical results of single CPC are not consistent with the theory. The largest single series of endoscopic CPC in history served as 82 patients in a stand-alone procedure, of which only 29 had a successful procedure (Pople and Ettles, 1995). The therapeutic effect of CPC alone is not as good as first thought, thus, the treatment of hydrocephalus with CPC alone has been basically eliminated. Recently, with the rise of endoscopic third ventriculostomy with CPC (ETV/CPC), CPC has been better applied in clinical treatment. Of course this is only 20 years (Warf, 2005). Therefore, it should be noted that the CPC in this paper is all part of ETV/CPC, rather than CPC alone. The probability of success of the ETV/CPC procedure was not inferior to that of ETV alone or CPC alone (Warf, 2005). The obvious benefit of ETV/CPC is that it does not depend on the shunt, which is one of the reasons for choosing ETV/CPC. For the selection of surgical subjects, it mainly depends on the age of the patient and the etiology. Patients younger than 1 year of age, patients with post-infectious hydrocephalus, and patients with partial or incomplete CPC were considered not eligible for ETV/CPC (Dewan and Naftel, 2017). Scar predicted ETV/CPC failure (Warf and Kulkarni, 2010; Stone and Warf, 2014), which suggests that CPC may not play a full role in the presence of a scar, is not effective in reducing CSF secretion but highlights the important role-played by ETV in ETV/CPC. For the question of the degree of CPC in ETV/CPC, there are studies indicating that the CP must be completely inactivated to achieve the best surgical results (Dewan and Naftel, 2017; Fallah et al., 2017). This may be due to the presence of compensatory CSF output in the CP; that is, a partially inactivated CP may lead to more CSF secretion by the remaining CP, resulting in poor surgical results. As an important part of the brain, whether a devastating blow to the CP can affect the patient's cognitive ability and other abilities after surgery has not been described, and further follow-up studies are needed to prove the utility of ETV/CPC and whether there are long-term side effects.

Overall, CPC now has a place in the treatment of hydrocephalus, and future functional studies that may be more precise for the CP may elicit the potential dangers of ETV/CPC surgery. The CP is an important part of CSF secretion by the brain and an important part in the pathogenesis of neurodegenerative diseases, yet it is incredible if the CP is directly burned so that it loses all relevant functions without minor side effects or even significant side effects. Choroid plexus cauterization inevitably results in abnormal recruitment of leukocytes by the brain, which will have an impact on the recovery of brain inflammation. As CSF-producing organs, the composition of CSF will change after inactivation of the choroid plexus, which will have a serious impact on the brain microenvironment, and will this increase the risk of other brain diseases such as AD in patients after choroid plexus burn surgery? It is hoped that future studies will answer if we have underestimated the compensatory function of the brain or not.

### Choroid plexus transplantation

Neurotrophic factors have shown good therapeutic effects for CNS diseases, but intravenous injection cannot be achieved due to the blood-brain barrier. In clinical practice, the supply of neurotrophic factors can only be used for intraventricular infusion using the instrument. This means that repeated injections are needed to maintain efficacy, and the implementation process is not convenient and simple, and there is even a risk of infection. Another idea is to implant genetically engineered cells to produce specific trophic factors. Although the use of this immortalized cell line can avoid many limitations with the form of mechanical delivery, the applicability of the vector, the stability of protein expression, and potential safety issues make the use of transgenic cells theoretically challenging. The CP, as an important component of CSF secretion, has a protective effect on neurons (Borlongan et al., 2004a). If a normal CP can be transplanted into the damaged brain site to allow the CP to secrete neurotrophic factors normally, it theoretically would have a certain therapeutic effect on the disease. Excitingly, the relevant studies conducted so far fully support the view. Choroid plexus transplantation has yielded exciting results in stroke, PD, and ischemic brain injury (Borlongan et al., 2004b; Emerich et al., 2004; Luo et al., 2013; Eslami et al., 2021).

The transplantation experiment of porcine choroid plexus brain has satisfactorily illustrated that choroid plexus transplantation has a significant protective effect on the structure and function of the brain in stroke animals, which coincides with our expectations (Borlongan et al., 2004b). In this study, the CP from neonatal pigs was artificially encapsulated in alginate microcapsules for neuroprotective studies *in vitro* and *in vivo*. Parallel *in vivo* studies have shown that CP transplantation improves behavioral performance, reduces infarct volume, and encapsulates microcapsules more significantly, even if neurotrophic factors produced by an encapsulated CP need to cross obstacles to play an important role. This is closely related to the ability of microcapsules to effectively prevent rejection of an encapsulated CP (Borlongan et al., 2004b). Future applications of choroid plexus transplantation in stroke patients should pay more attention to the choice of transplantation site and the mode of choroid plexus transplantation, and we can do more research to find if there is a more effective treatment than encapsulation in alginate microcapsules.

Some studies have used neonatal porcine choroid plexus to transplant into PD rhesus monkeys, and because the cerebrospinal fluid secreted by the CP contains various neurotrophic and neuroprotective factors, the researchers hope that choroid plexus transplantation can improve parkinsonian symptoms in immunosuppressed rhesus monkeys treated with MPTP (1-methyl-4-phenyl-1,2,3,6-tetrahydropyridine). The findings were unequivocal, with a significant improvement in neurological function in MPTP-treated monkeys within 6 months after transplantation compared to monkeys implanted with empty capsules or subjected to sham surgery. Improvements in neurological scores were accompanied by a corresponding improvement in apomorphine-induced circling behavior and increased striatal tyrosine hydroxylase (TH) staining, all of which indicate that choroid plexus transplantation plays an important role in the treatment of PD monkeys. Because the caudate nucleus of the striatum is close to the ventricles and can be easily identified from computed tomography (CT) cross-sectional images, the caudate nucleus of the striatum was selected as the transplantation site (Luo et al., 2013). Experiments demonstrated that implanting a CP known to secrete many neuroprotective and neuro repair factors into the injured site of a primate model of PD restored striatal nerve fiber networks and improved neuro behavior in animals. The results are consistent with previous studies demonstrating the neuro repair function exhibited by a porcine CP in various animal models of neurodegenerative diseases. This showed a potential value of choroid plexus transplantation for the treatment of PD in primates.

Researchers examined the ability of a cultured CPE to protect against ischemic brain injury when transplanted into rat CSF. In the experiment, the rat's middle cerebral artery was transiently occluded, and then the researchers injected an artificially cultured CPE into the fourth ventricle. Injection significantly reduced the neurological deficit and infarct volume within 24 h. These included reduced numbers of apoptotic and inflammatory cells in the brain, upregulated mRNA expression of the anti-apoptotic effector camp response element-binding protein, and downregulated production of proinflammatory factors such as interleukin-1β and inducible nitric oxide lyase (Matsumoto et al., 2010). It is worth noting that the injected CPEs in the experiment were located in the ventricle and on the surface of the brain and were not ischemic foci, which indeed indicates that they can play their role in the CSF by releasing diffusible neuroprotective factors. It directly illustrates that transplantation of CPEs *via* CSF is a potential new approach to protect against ischemic brain injury.

**Figure 3:**
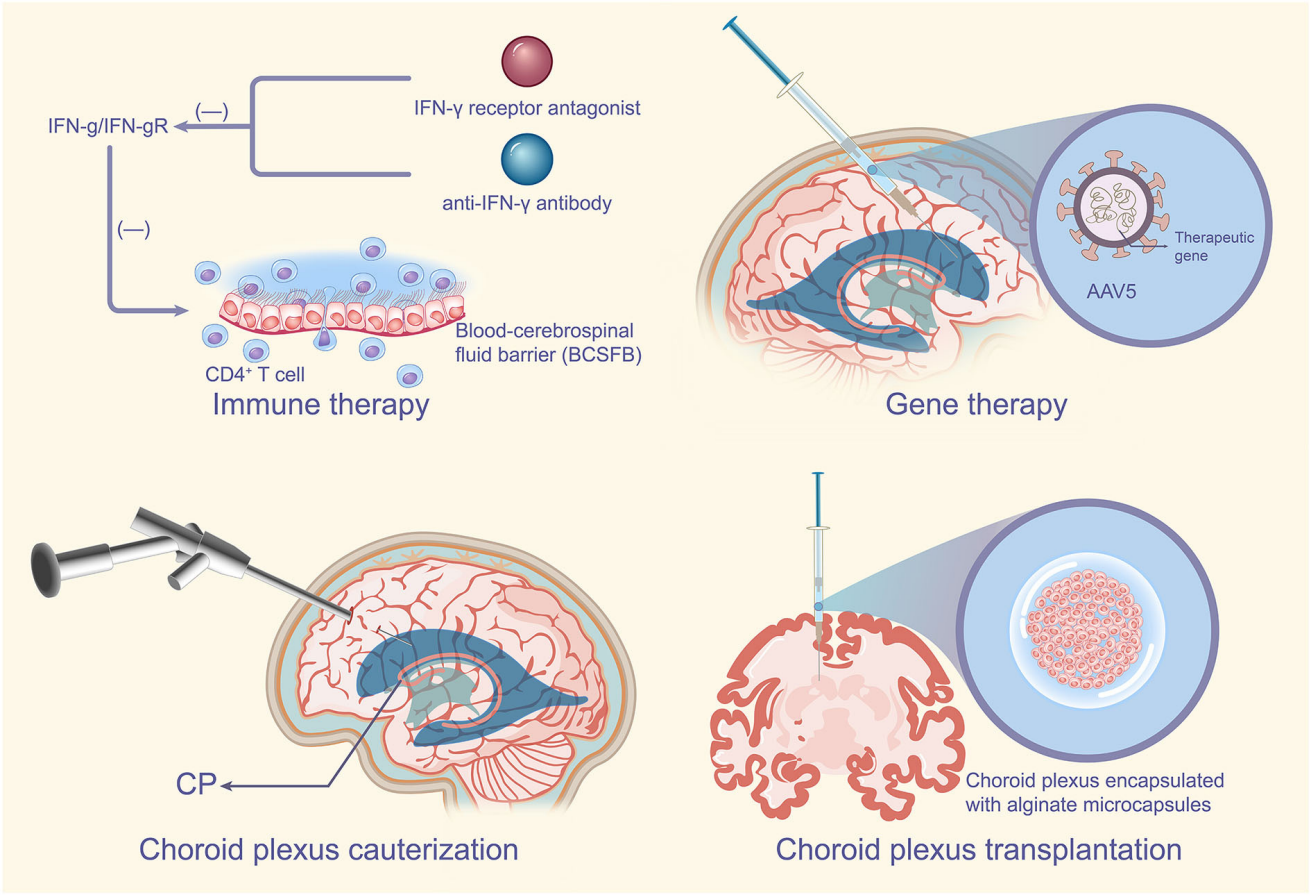
CPE-based therapeutic measures. For autoimmune disease MS, specific blockade of the IFN-γ signaling pathway in the CP such as the addition of IFN-γ receptor antagonists or anti-IFN-γ antibodies may improve the development of MS. Targeted delivery of certain effective therapeutic genes through the specific recognition of the CP by AAV5 has very important therapeutic implications for neurodegenerative diseases of the brain or some refractory diseases. CPC can reduce CSF secretion by the CP, which has no doubts about the therapeutic significance for the CP, of course CPC is usually used in combination with ETV. Transplantation of the choroid plexus encapsulated in special containers such as alginate microcapsules into the affected brain can improve pre-existing neuroinflammation with potential implications for the treatment of PD. MS, multiple sclerosis; IFN-γ, interferon-γ; CP, choroid plexus; AAV5, adeno-associated virus type 5; CPC, choroid plexus cauterization; CSF, cerebrospinal fluid; ETV, endoscopic third ventriculostomy; PD, Parkinson's disease.

In conclusion, choroid plexus transplantation has demonstrated good therapeutic effects in many models of inflammatory diseases or neurodegenerative diseases of the brain (Borlongan et al., 2004b; Luo et al., 2013). Transplantation of porcine choroid plexus encapsulated with special containers such as alginate microcapsules into the affected brain does improve pre-existing neuroinflammation (Borlongan et al., 2004b; Emerich et al., 2004). However, the target object and mode of transplantation can be more perfect, and the indications of transplantation need further study (Figure 3).

## Conclusions

As the main site of CSF secretion in the brain, the CP is also involved in the regulation of the neurogenesis circadian clock, the transmission of inflammatory signals and the important role in atypical neurodevelopmental processes. The function of the CP has been described by many studies, and the tight junctions on it are important structures that constitute the blood-cerebrospinal fluid barrier.

CSF secreted by the CP plays a role in regulating neurodevelopment and nourishing nerves, and CSF can provide a stable environment for the brain. The description of accelerated atrophy of the CP in stroke, multiple sclerosis, schizophrenia, and other central nervous system diseases illustrates that atrophy of the CP is associated with CNS diseases, and the relationship between ciliary abnormalities on the CPE and hydrocephalus is also illustrated. There must be a close link between CP pathology and cognitive and neurodegenerative diseases. The role of the CP in preventing neurological diseases includes maintaining tight junctions in the CPE to maintain the blood-cerebrospinal fluid barrier, promoting CSF circulation as well as promoting the excretion of waste products from CSF.

Therapeutic modalities with the CP as a therapeutic target include immunotherapy, gene therapy, choroid plexus cauterization, as well as choroid plexus transplantation. The above treatment modalities have shown great value for the treatment of neurological diseases and are worthy of further study. The author believes that the CP can be used as a good viral vector for the purpose of gene therapy. Due to its great advantages of crossing the blood-brain barrier, the author believes that if this is used rationally, it may bring unexpected breakthroughs in the treatment of neurological diseases.

The profound understanding of the CP enables us to view neurological diseases from a new perspective that is CP-centered, even if the etiology of these diseases is not single. As a CP that produces the ecological environment in the brain, it is not too much to pay attention to how it is actually.

## Author contributions

YC participated in the drawing of the figure of the article. ZZ, YC, JL, and YW participated in the collection and revision of the article. ZL participated in the polishing of the article. All authors read and approved the final manuscript.

## Funding

This work was supported by National Natural Science Foundation of China (No. 82171347) and the Students Innovations in Central South University of China (Nos. 20210033020055, 20210033020036, and 20210033020044).

## Conflict of interest

The authors declare that the research was conducted in the absence of any commercial or financial relationships that could be construed as a potential conflict of interest. The reviewer QC declared a past co-authorship with the author GX to the handling editor.

## Publisher's note

All claims expressed in this article are solely those of the authors and do not necessarily represent those of their affiliated organizations, or those of the publisher, the editors and the reviewers. Any product that may be evaluated in this article, or claim that may be made by its manufacturer, is not guaranteed or endorsed by the publisher.

## Abbreviations

CPE: choroid plexus epithelium
CNS: central nervous system
BCSFB: blood-cerebrospinal fluid barrier
CSF: cerebrospinal fluid
AD: Alzheimer's disease
TJ: tight junction
γ-Pcdh: Pcdh-γ gene family
CP: choroid plexus
SCN: suprachiasmatic nucleus
Aβ: amyloid-β
PHH: posthemorrhagic hydrocephalus
TLR4: toll-like receptor 4
IVH: intraventricular hemorrhage
ZT-1A: 5-chloro-N-(5-chloro-4-((4-chlorophenyl)(cyano)methyl)-2-methylphenyl)-2-hydroxybenzamide
CCC: cation-Cl^−^ cotransporters
LRP-1 and 2: lipoprotein receptor-related proteins 1 and 2
MMP: matrix metalloproteinases
PD: Parkinson's disease
DA: dopamine
Cp: ceruloplasmin
CPEC-CM: choroid plexus epithelial cell conditioned medium
KS: knockout serum
hADSCs: human adipose-derived stem cells
PACRG: parkin co-regulated gene
MS: multiple sclerosis
IFN-α: interferon-α
IFN-γ: interferon-γ
MRI: magnetic resonance imaging
IFN-β: interferon-β
EVA: epithelial v-like antigen
AAV: adeno-associated virus
AAV5: adeno-associated virus type 5
TLR9: toll-like receptor 9
CPC: choroid plexus cauterization
ETV/CPC: endoscopic third ventriculostomy with CPC
TH: tyrosine hydroxylase
CT: computed tomography.
